# Hypoxia-targeted ^131^I therapy of hepatocellular cancer after systemic mesenchymal stem cell-mediated sodium iodide symporter gene delivery

**DOI:** 10.18632/oncotarget.10758

**Published:** 2016-07-21

**Authors:** Andrea M. Müller, Kathrin A. Schmohl, Kerstin Knoop, Christina Schug, Sarah Urnauer, Anna Hagenhoff, Dirk-André Clevert, Michael Ingrisch, Hanno Niess, Janette Carlsen, Christian Zach, Ernst Wagner, Peter Bartenstein, Peter J. Nelson, Christine Spitzweg

**Affiliations:** ^1^ Department of Internal Medicine II, University Hospital of Munich, Ludwig Maximilian University of Munich, Munich, Germany; ^2^ Clinical Biochemistry Group, Medizinische Klinik und Poliklinik IV, University Hospital of Munich, Ludwig Maximilian University of Munich, Munich, Germany; ^3^ Department of Clinical Radiology, University Hospital of Munich, Ludwig Maximilian University of Munich, Munich, Germany; ^4^ Department of General, Visceral, Transplantation, Vascular and Thoracic Surgery, University Hospital of Munich, Ludwig Maximilian University of Munich, Munich, Germany; ^5^ Department of Nuclear Medicine, University Hospital of Munich, Ludwig Maximilian University of Munich, Munich, Germany; ^6^ Department of Pharmacy, Center of Drug Research, Pharmaceutical Biotechnology, Ludwig Maximilian University of Munich, Munich, Germany

**Keywords:** hypoxia-targeting, sodium iodide symporter, mesenchymal stem cells, hepatocellular carcinoma, gene therapy

## Abstract

Adoptively transferred mesenchymal stem cells (MSCs) home to solid tumors. Biologic features within the tumor environment can be used to selectively activate transgenes in engineered MSCs after tumor invasion. One of the characteristic features of solid tumors is hypoxia. We evaluated a hypoxia-based imaging and therapy strategy to target expression of the sodium iodide symporter (NIS) gene to experimental hepatocellular carcinoma (HCC) delivered by MSCs.

MSCs engineered to express transgenes driven by a hypoxia-responsive promoter showed robust transgene induction under hypoxia as demonstrated by mCherry expression in tumor cell spheroid models, or radioiodide uptake using NIS. Subcutaneous and orthotopic HCC xenograft mouse models revealed significant levels of perchlorate-sensitive NIS-mediated tumoral radioiodide accumulation by tumor-recruited MSCs using ^123^I-scintigraphy or ^124^I-positron emission tomography. Functional NIS expression was further confirmed by *ex vivo* ^123^I-biodistribution analysis. Administration of a therapeutic dose of ^131^I in mice treated with NIS-transfected MSCs resulted in delayed tumor growth and reduced tumor perfusion, as shown by contrast-enhanced sonography, and significantly prolonged survival of mice bearing orthotopic HCC tumors. Interestingly, radioiodide uptake into subcutaneous tumors was not sufficient to induce therapeutic effects. Our results demonstrate the potential of using tumor hypoxia-based approaches to drive radioiodide therapy in non-thyroidal tumors.

## INTRODUCTION

Hepatocellular carcinoma (HCC) is the most common form of liver cancer, and the third most frequent cause of cancer-related death worldwide [1]. HCC is usually diagnosed at advanced stages which limits therapeutic options as only a fraction of HCC patients are candidates for surgical resection [2, 3]. Therefore, there is an urgent need for novel therapeutic approaches to cure or control HCC growth.

Solid tumors, including HCCs, are composed of malignant tumor cells within a “benign” stromal environment containing hepatic stellate cells, cells of the immune system, smooth muscle cells, endothelial cells and pericytes/cancer-associated fibroblasts (CAFs) [4, 5]. The malignant phenotype of a tumor not only depends on autonomous properties of cancer cells, but also on crosstalk between the tumor cells and the stromal compartment [4, 6]. Because the tumor stroma plays such a major role in tumor progression, it represents an important target for tumor therapy.

Mesenchymal stem cells (MSCs) play key roles in the maintenance and regeneration of diverse tissues based on their ability to differentiate into cells of connective tissue lineages [4]. Upon tissue injury or during chronic inflammation, MSCs are recruited to these sites where they contribute to tissue remodeling [4, 7]. Tumors have been described as “wounds that never heal” [8], driving continuous tissue remodeling with pronounced recruitment and proliferation of MSCs [7]. This has led to the investigation of MSCs for use as shuttle vectors for the delivery of therapeutic agents deep into growing tumors. In our previous preclinical studies using the “suicide gene” herpes simplex virus type 1 thymidine kinase (HSV-TK) [9–11] or the sodium iodide symporter (NIS) [12–14] as therapy genes, we demonstrated the active homing of MSCs to the tumor stroma resulting in a significant reduction in tumor growth and prolonged animal survival after the application of ganciclovir or ^131^I, respectively. This work was used to help develop a prospective phase I/II clinical trial for treatment of advanced, recurrent or metastatic gastrointestinal or hepatopancreatobiliary adenocarcinoma using autologous genetically engineered MSCs expressing the HSV-TK gene [15].

NIS may represent a better therapy gene for future trials based on its dual function as reporter and therapy gene. As a reporter gene, it allows non-invasive imaging of tumoral MSC recruitment and biodistribution as well as duration and level of NIS transgene expression by ^99m^Tc-/^123^I-scintigraphy, ^123^I-SPECT (single photon emission computed tomography) or ^124^I-/^18^F-tetrafluoroborate-PET (positron emission tomography) and thus exact dosimetric calculations before the application of therapeutic radionuclides such as ^131^I or ^188^Re [16–18].

We and others have demonstrated that adoptively transferred MSCs are recruited to normal tissues as part of tissue homeostasis [19]. Therefore, it is important to limit expression of the therapy gene to the tumor environment in order to prevent potential undesired side effects. This can be achieved by enhancing the tumor specificity of transgene transduction through the use of gene promoters that are selectively activated in the engineered MSCs when they contact the tumor environment.

In previous preclinical studies we made use of the RANTES gene promoter for enhanced tumor-targeting of MSC-mediated NIS expression. This was based on the observation that the RANTES/CCL5 chemokine is induced by MSCs following their recruitment to the tumor stroma and differentiation into CAFs [13, 14]. The RANTES-mediated targeting led to significant radioiodide accumulation in subcutaneous HCC xenografts after the systemic injection of MSCs engineered to express NIS under control of the RANTES promoter (RANTES-NIS-MSC). Treatment led to a delay in tumor growth and improved survival after the application of a therapeutic dose of ^131^I [13].

Tumor hypoxia represents an important challenge in cancer therapy. It results from the rapid proliferation rate of solid tumors causing an outstripping of the oxygen supply provided by the local vasculature [20, 21]. Hypoxia-inducible factor (HIF)-1 is the key mediator of the cellular response to hypoxia, activating the expression of multiple genes that participate in key aspects of tumor progression [21–23]. Compared to normoxic tumor cells, hypoxic tumor cells are more resistant to conventional treatment options such as chemo- or radiotherapy and tumor hypoxia is associated with a more malignant phenotype [24, 25]. Therefore, selective targeting of hypoxic tumor cells has become a central issue in cancer therapy. In the present study, we evaluated the use of MSCs with a synthetic HIF-responsive promoter to target functional NIS expression into a subcutaneous and an orthotopic HCC xenograft mouse model.

## RESULTS

### *In vitro* characterization of MSCs stably expressing reporter genes controlled by a synthetic HIF-responsive promoter

**Figure 1:**
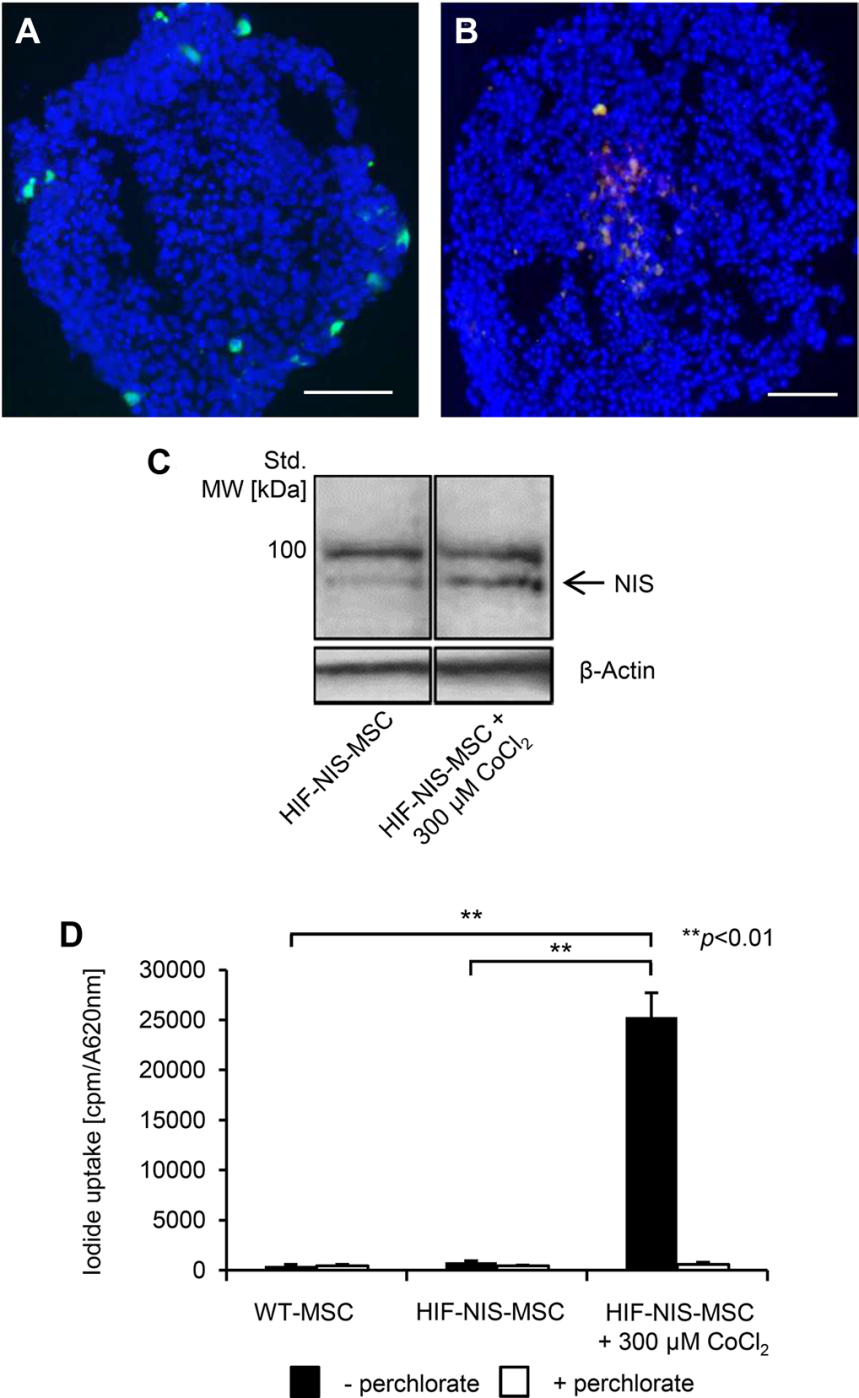
Induction of hypoxia-responsive promoter-driven transgene expression under hypoxic conditions *in vitro* (**A, B**) For invasion assays, MSCs were stably transfected with mCherry controlled by a hypoxia-responsive promoter (HIF-Cherry-MSCs) and labeled with a green fluorescent cell tracker dye. HIF-Cherry-MSCs located at the surface and thus in non-hypoxic areas of the HuH7 cell spheroid appear green (A), while after invasion into hypoxic areas of spheroids, HIF-Cherry-MSCs appear orange due to an overlay of hypoxia-induced mCherry expression and the green fluorescent cell tracker dye (B). Scale bar = 100 μm. (**C**) Western blot analysis of MSCs stably transfected with NIS driven by a hypoxia-responsive promoter (HIF-NIS-MSCs) showed increased NIS protein expression with a major band of ~90 kDa after stimulation with the hypoxia mimicking agent cobalt chloride as compared to unstimulated HIF-NIS-MSCs. Cropped blots are shown. MW, molecular weight; Std., standard. (**D**) ^125^I uptake studies revealed a 31-fold increased perchlorate-sensitive NIS-mediated radioiodide uptake in HIF-NIS-MSCs after stimulation with cobalt chloride as compared to unstimulated HIF-NIS-MSCs. WT-MSCs and unstimulated HIF-NIS-MSCs showed no perchlorate-dependent ^125^I accumulation above background level. Results are reported as mean ± SEM (*n* = 3; **p < 0.01).

To analyze the inducibility of the synthetic HIF-responsive promoter, an experimental three-dimensional HCC cell (HuH7) spheroid model was used to mimic the hypoxia seen in tumors. Spheroid models show a hypoxic gradient that forms after the first few cell layers of the spheroid and is maximal at the center of the structure. Human MSCs (WT-MSCs) stably transfected with the red fluorescent protein mCherry under the control of the HIF-responsive promoter (HIF-Cherry-MSCs) were also labeled with the green fluorescent dye CMFDA. After invasion into the hypoxic center of HuH7 spheroids, the HIF-Cherry-MSCs showed induced transgene expression. As MSCs were labeled with a green fluorescent dye, cells appear orange after mCherry expression due to overlay of red and green fluorescence signals (Figure 1B), while MSCs at the non-hypoxic borders of spheroids do not express mCherry and appear green (Figure 1A).

As a next step, WT-MSCs were engineered to express NIS under the control of the HIF-responsive promoter (HIF-NIS-MSCs). The HIF-NIS-MSCs showed increased NIS protein expression after 24 h stimulation with the hypoxia-simulating agent cobalt chloride, as confirmed by Western blot (Figure 1C). In a functional assay, HIF-NIS-MSCs showed a 31-fold higher radioiodide uptake when treated with cobalt chloride as compared to untreated HIF-NIS-MSCs. NIS-mediated radioiodide uptake was blocked by the competitive NIS inhibitor sodium perchlorate. Untreated HIF-NIS-MSCs and WT-MSCs showed no ^125^I uptake above background level (Figure 1D).

### *In vivo* radioiodide biodistribution imaging after MSC application

**Figure 2:**
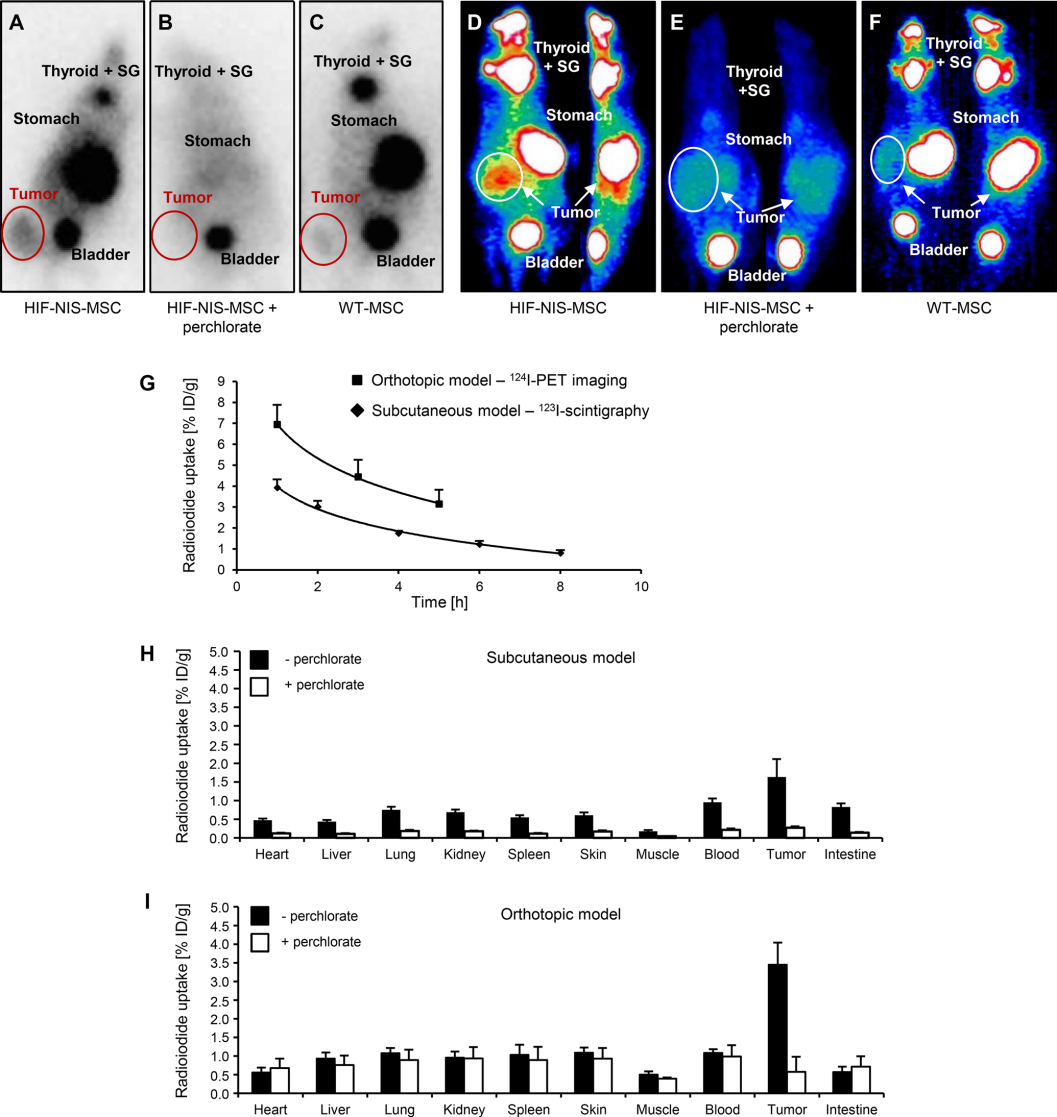
Enhanced tumoral radioiodide accumulation after systemic HIF-NIS-MSC application in subcutaneous and intrahepatic HuH7 xenograft mouse models ^123^I-scintigraphy (**A–C**) or ^124^I-PET (**D–F**) imaging demonstrated enhanced radioiodide accumulation in subcutaneous (A; *n* = 12) and orthotopic (D; *n* = 6) HCC tumors after systemic HIF-NIS-MSC application 3 h after radioiodide injection, which was blocked by the competitive NIS inhibitor sodium perchlorate (B, E; subcutaneous model: *n* = 4; orthotopic model: *n* = 3), while mice injected with WT-MSC showed no tumoral radioiodide uptake above background level (C, F; subcutaneous model: *n* = 9; orthotopic model: *n* = 2). One representative image is shown per group. (**G**) Time course of radioiodide accumulation in HuH7 tumors as determined by serial scanning. Subcutaneous HCC xenografts showed a maximum ^123^I uptake of 3.9 ± 0.4% ID/g with a biological half-life of 3.8 h, whereas orthotopic HCC tumors accumulated up to 6.9 ± 0.9% ID/g ^124^I with a biological half-life of 4.0 h. Results are reported as mean ± SEM. *Ex vivo* biodistribution analysis confirmed perchlorate-sensitive radioiodide accumulation in subcutaneous ((**H**); 1.6 ± 0.5% ID/g; *n* = 12; perchlorate: *n* = 5) and intrahepatic ((**I**); 3.5 ± 0.6% ID/g; *n* = 6; perchlorate: *n* = 3) tumors. No significant radioiodide accumulation was measured in non-target organs. Results are reported as mean ± SEM.

To assess MSC-mediated delivery of hypoxia-induced NIS expression *in vivo,* nude mice harboring subcutaneous HCC xenograft tumors were injected with HIF-NIS-MSCs or WT-MSCs three times via the tail vein followed by one intraperitoneal ^123^I injection 72 h later. Radioiodide biodistribution assessed using ^123^I-gamma camera imaging revealed a tumoral radioiodide uptake of 3.9 ± 0.4% of the total amount of the applied ^123^I dose per gram tumor (% ID/g) with a biological half-life of 3.8 h (Figure 2A, 2G). A tumor-absorbed dose of 26.5 mGy/MBq/g tumor ^131^I with an effective half-life of 3.8 h was calculated. Mice treated with WT-MSCs showed no tumoral radioiodide accumulation above background level (Figure 2C). The radioiodide uptake observed in the thyroid gland, the salivary glands (SG) and in the stomach results from endogenous NIS expression. Radioiodide accumulation in the urinary bladder is due to renal excretion of the radionuclide (Figure 2A–2F). To confirm that tumoral radioiodide accumulation was NIS-mediated, a subgroup of animals received the NIS-specific inhibitor sodium perchlorate 30 min before ^123^I injection, which resulted in strongly reduced radioiodide uptake in tumor, stomach, thyroid and SG (Figure 2B).

In the next step, our hypoxia-based strategy was examined in a more clinically relevant orthotopic HCC xenograft mouse model. Human HCC cells (HuH7) were injected directly into the liver of nude mice, leading to solid intrahepatic xenograft tumors. Nude mice bearing the orthotopic xenografts were injected with three rounds of HIF-NIS-MSCs or WT-MSCs via the tail vein. 72 h after the last MSC injection, 13 MBq ^124^I were applied intraperitoneally and radioiodide accumulation in the intrahepatic xenografts was determined by small-animal PET imaging. One hour after ^124^I injection, HCC xenografts had accumulated 6.9 ± 0.9% ID/g with a biological half-life of 4.0 h (Figure 2D, 2G). Dosimetric calculations revealed a tumor-absorbed dose of 46.8 mGy/MBq/g tumor with an effective half-life of 3.9 h for ^131^I. Tumoral radioiodide accumulation was found to be strongly reduced after a single injection of sodium perchlorate, as were stomach, thyroidal and SG uptake (Figure 2E). In WT-MSC-injected mice no significant radioiodide uptake was detected (Figure 2F).

### *Ex vivo* radioiodide biodistribution analysis after MSC application

To quantify HIF-NIS-MSC-mediated tumoral radioiodide accumulation in the subcutaneous and intrahepatic HCC tumors, *ex vivo* ^123^I-biodistribution analysis was performed 4 h after radioiodide injection by measuring the ^123^I uptake in individual tissues. The results showed a perchlorate-sensitive radioiodide uptake of 1.6 ± 0.5% ID/g in subcutaneous tumors (Figure 2H) and 3.5 ± 0.6% ID/g in intrahepatic tumors (Figure 2I), respectively. No ^123^I accumulation above background level was seen in non-target organs (Figure 2H, 2I).

### Immunohistochemical analysis of MSC biodistribution and NIS expression

**Figure 3:**
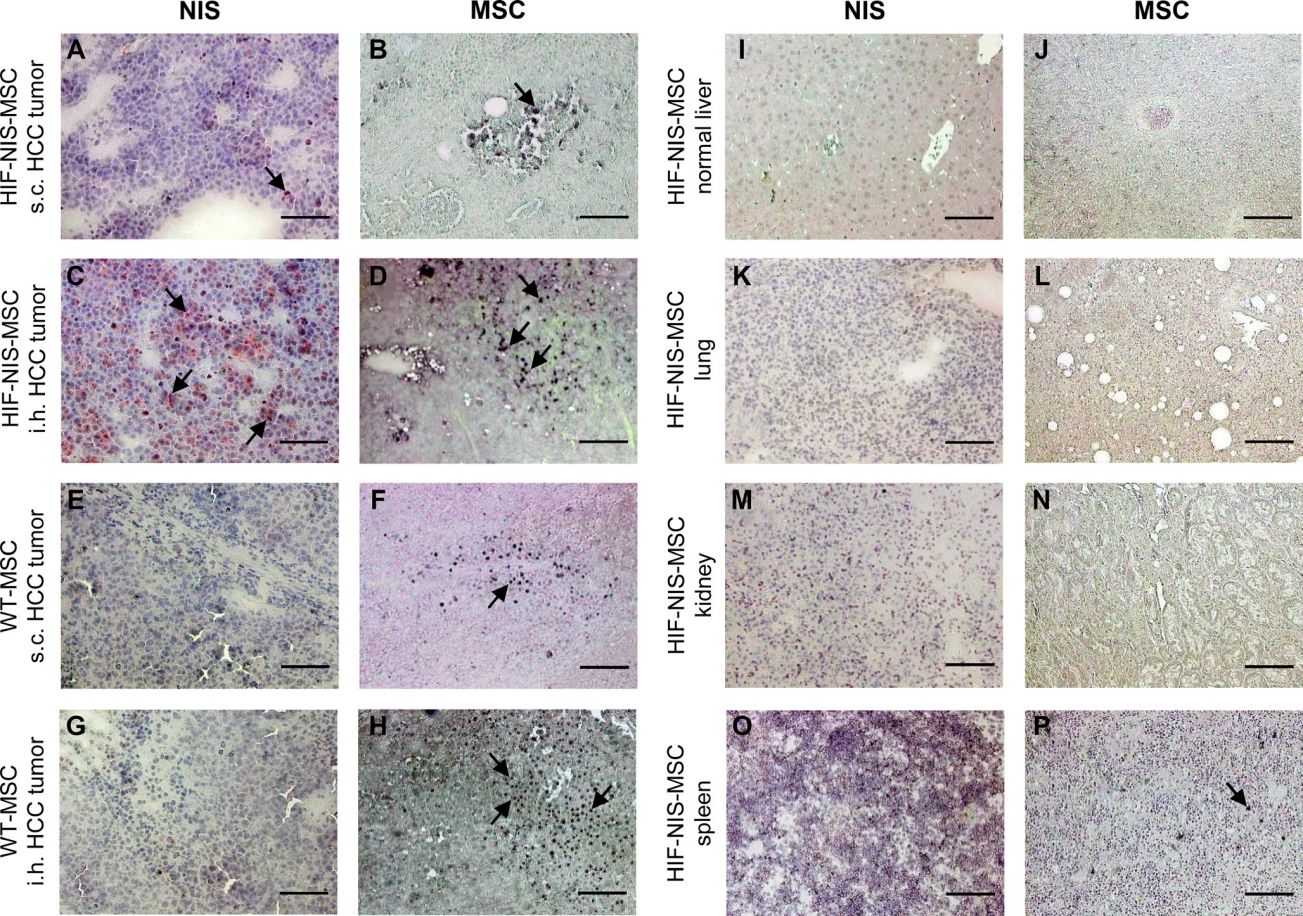
MSC recruitment and hypoxia-induced NIS expression were higher in intrahepatic compared to subcutaneous HCC tumors Compared to subcutaneous (s.c.) tumors (**A**), higher NIS-specific immunoreactivity was detected in intrahepatic (i.h.) HuH7 tumors (**C**). This correlated well with tumoral HIF-NIS-MSC recruitment (**B, D**). In mice injected with WT-MSCs, no NIS expression (**E, G**) was detected, though MSCs were recruited (**F, H**). Non-target organs showed neither MSC recruitment nor NIS expression (**I–N**), except for the spleen where no NIS staining (**O**) but positive MSC staining (**P**) were observed. One representative image is shown each. Scale bar = 100 μm.

To determine MSC biodistribution within the tumor and non-target organs (lung, normal liver, kidney and spleen), tissue sections were stained for the presence of the human MSCs or NIS protein expression after ^123^I-scintigraphy or ^124^I-PET imaging, respectively. Staining of subcutaneous xenografts revealed only weak tumor stromal NIS expression (Figure 3A) and relatively low MSC recruitment (Figure 3B). In comparison, orthotopic HuH7 tumors showed high levels of hypoxia-induced NIS protein expression in the tumor stroma (Figure 3C), but not in the surrounding normal liver tissue (Figure 3I). Further, high levels of MSCs were detected in the stroma of orthotopic liver tumors (Figure 3D). In both tumor models, WT-MSC-injected mice showed MSC recruitment with no NIS immunoreactivity (Figure 3E–3H). In non-target organs of both tumor models, neither MSC immunoreactivity nor NIS immunostaining were detected (Figure 3I–3N), with the exception of the spleen, where MSCs were detected (Figure 3P) but no NIS expression was observed (Figure 3O).

### Radioiodide therapy of subcutaneous and orthotopic HCCs after MSC-mediated NIS gene transfer

**Figure 4:**
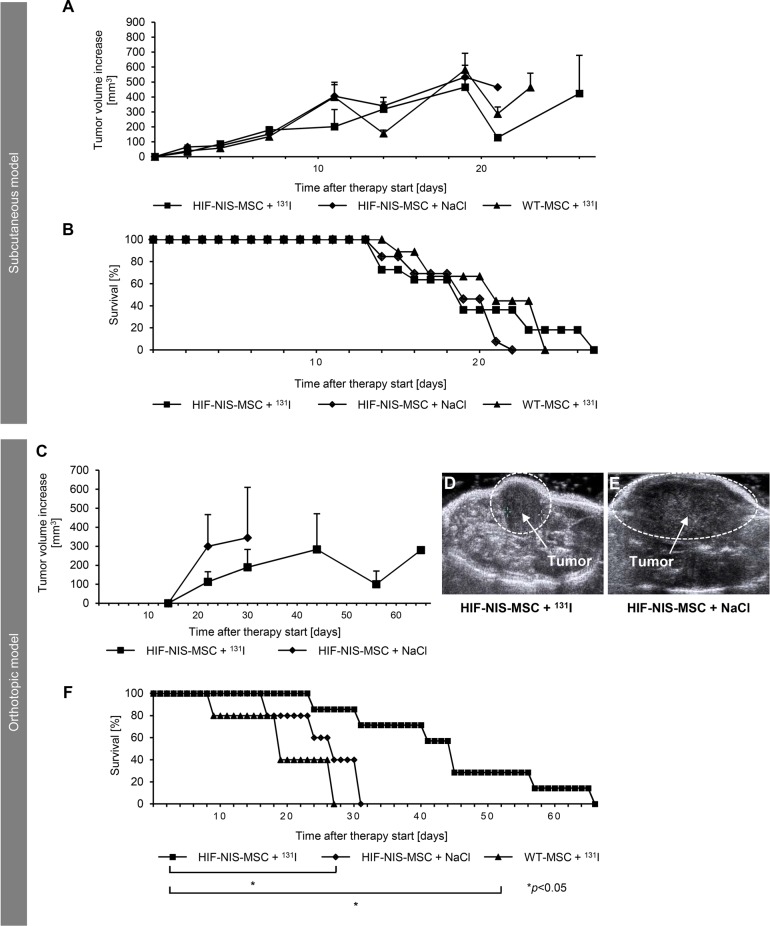
Growth inhibition of orthotopic HuH7 tumors after application of a therapeutic dose of radioiodide in HIF-NIS-MSC-treated mice was associated with a prolonged survival Two groups of mice were established that received 55.5 MBq ^131^I 48 h after the final of three HIF-NIS-MSC (subcutaneous model: *n* = 11; orthotopic model: *n* = 7) or WT-MSC (subcutaneous model: *n* = 9; orthotopic model: *n* = 5) applications in 2-day-intervals. This cycle was repeated once 24 h after the last radioiodide application. 24 h after these treatment cycles, one additional MSC injection was administered followed by a third ^131^I injection 48 h later. A further control group received HIF-NIS-MSCs and NaCl (subcutaneous model: *n* = 13; orthotopic model: *n* = 5). In animals harboring subcutaneous HCC xenografts, no significant difference in tumor growth ((**A**); mean ± SEM) or animal survival ((**B**); percent survival) was observed comparing therapy to control animals (WT-MSC + ^131^I and HIF-NIS-MSC + NaCl). In mice harboring orthotopic HuH7 tumors, HIF-NIS-MSC/^131^I application resulted in reduced tumor growth ((**C–E**); mean ± SEM) and increased mouse survival ((**F**); percent survival) as compared to control groups (WT-MSC + ^131^I; *p < 0.05 and HIF-NIS-MSC + NaCl; *p < 0.05).

The therapeutic effect of ^131^I was assessed in mice bearing subcutaneous or orthotopic HCCs using a therapy scheme established in previous studies [12, 13]. After tumor establishment, mice in the therapy group received three cycles of intravenous HIF-NIS-MSC applications followed by intraperitoneal ^131^I injections, while control mice received either HIF-NIS-MSCs followed by saline or WT-MSCs followed by ^131^I. In mice harboring subcutaneous HCC tumors, no significant difference in tumor growth (Figure 4A) or animal survival (Figure 4B) was observed between the therapy group and the control groups (HIF-NIS-MSC + NaCl and WT-MSCs + ^131^I). By contrast the orthotopically implanted HuH7 xenografts were better targeted by the hypoxia signal. Growth of orthotopic HuH7 xenografts was monitored by contrast-enhanced ultrasonography (CEUS) starting from day 14 after therapy start. In the therapy group, slower tumor growth (Figure 4C–4E) and a significantly improved survival of up to nearly nine weeks were observed as compared to control groups (HIF-NIS-MSC + NaCl and WT-MSCs + ^131^I) which survived only up to four weeks (Figure 4F) after start of therapy.

**Figure 5:**
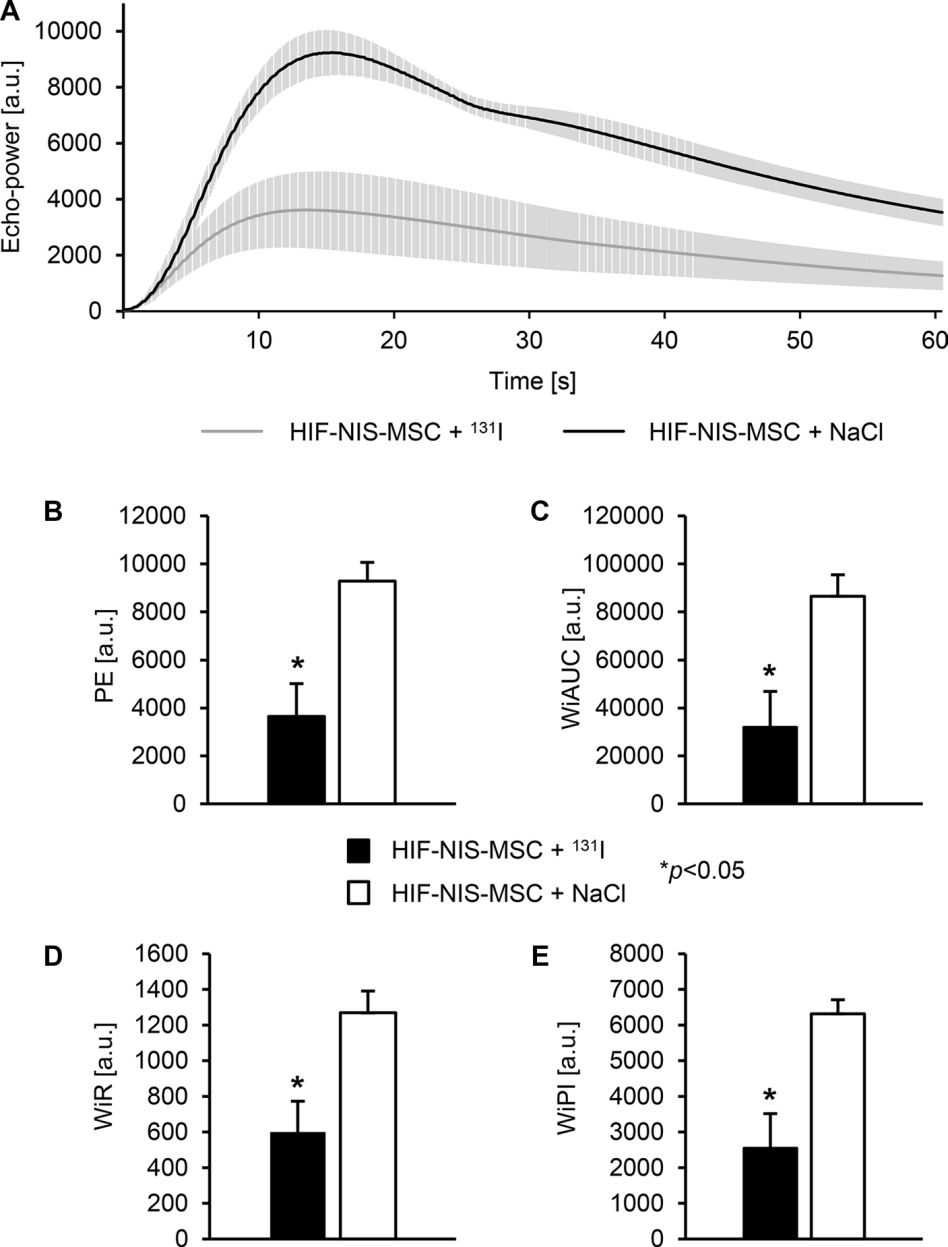
Reduced perfusion of intrahepatic HCC tumors after application of a therapeutic dose of radioiodide in HIF-NIS-MSC-treated mice Orthotopic HuH7 xenografts of HIF-NIS-MSC-treated mice showed an overall reduced contrast agent signal (**A**), PE (3652.6 ± 1364.0; (**B**)), WiAUC (32089.9 ± 14842.2; (**C**)), WiR (600.4 ± 172.2; (**D**)) and WiPI (2556.7 ± 960.5; (**E**)) after radioiodide therapy as compared to animals treated with HIF-NIS-MSC and NaCl (PE: 9281.9 ± 674.0; WiAUC: 86506.3 ± 7779.2; WiR: 1269.1 ± 106.0; WiPI: 6315.4 ± 342.3; (**B**–**E**)). Results are expressed as mean ± SEM in arbitrary units (a.u.; *p < 0.05).

For the assessment of tumor perfusion, echo signals from orthotopic HuH7 xenografts were measured over 1 min six days after the end of therapy using CEUS. Mice of the control group (HIF-NIS-MSC + NaCl) showed an overall increased signal from the contrast agent and a higher maximum signal as compared to the therapy group (Figure 5A). Reduced echo intensity in tumors of therapy mice was associated with significantly reduced peak enhancement (PE; 3652.6 ± 1364.0; Figure 5B), wash-in area under the curve (WiAUC; 32089.9 ± 14842.2; Figure 5C), wash-in rate (WiR; 600.4 ± 172.2; Figure 5D) and wash-in perfusion index (WiPI; 2556.7 ± 960.5; Figure 5E) compared to tumors of control mice (PE: 9281.9 ± 674.0; WiAUC: 86506.3 ± 7779.2; WiR: 1269.1 ± 106.0; WiPI: 6315.4 ± 342.3; Figure 5B–5E).

**Figure 6:**
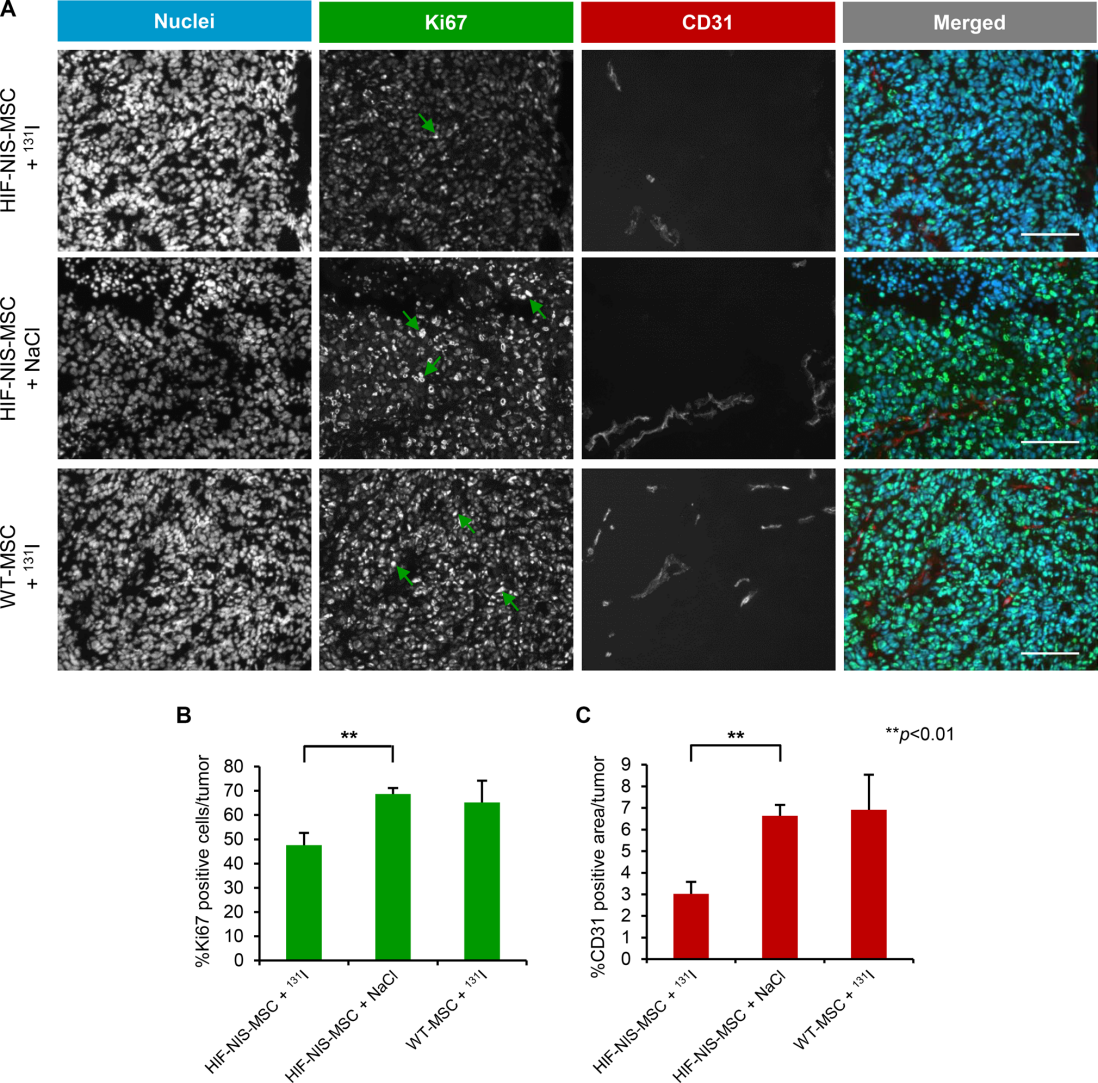
Reduced cell proliferation and blood vessel density in intrahepatic HCC tumors after application of a therapeutic dose of radioiodide in HIF-NIS-MSC-treated mice Orthotopic HuH7 tumors of HIF-NIS-MSC-treated mice demonstrated significantly reduced tumor cell proliferation (green in (**A**); 47.6 ± 5.0%; (**B**)) and blood vessel density (red in (**A**); 3.0 ± 0.5%; (**C**)) after radioiodide therapy as compared to animals treated with HIF-NIS-MSCs and NaCl ((**A**–**C**); Ki67: 68.7 ± 2.5%; CD31: 6.6 ± 0.5%) or WT-MSCs and ^131^I ((**A**–**C**); Ki67: 65.2 ± 9.0%; CD31: 6.9 ± 1.6%). Nuclei were counterstained with Hoechst dye. Results are expressed as mean ± SEM (**p < 0.01). Scale bar = 100 μm.

To evaluate cell proliferation (Ki67; green in Figure 6) and blood vessel density (CD31; red in Figure 6) after therapy, intrahepatic HCCs were analyzed by immunofluorescence staining. In mice with intrahepatic tumors the application of HIF-NIS-MSCs followed by ^131^I resulted in significantly reduced proliferation (47.6 ± 5.0%; Figure 6A, 6B) and blood vessel density (3.0 ± 0.5%; Figure 6A, 6C) as compared to animals treated with HIF-NIS-MSCs and NaCl (Ki67: 68.7 ± 2.5%; CD31: 6.6 ± 0.5%; Figure 6A–6C) or WT-MSCs and ^131^I (Ki67: 65.2 ± 9.0%; CD31: 6.9 ± 1.6%; Figure 6A–6C).

## DISCUSSION

As the microenvironment within solid tumors is similar to that seen in chronically injured tissues, solid tumors offer a permissive environment for the efficient engraftment of exogenously applied MSCs [7]. Hence, MSCs have emerged as potential cellular vehicles for the targeted delivery of therapeutic genes deep into the tumor microenvironment [26]. This approach has been explored in various preclinical cancer models yielding potent anti-tumor effects [4, 27]. In a series of previous studies we demonstrated active homing of HSV-TK-transfected MSCs into pancreatic, breast and liver tumor stroma that resulted in significantly reduced tumor growth and lower incidence of metastases after application of ganciclovir [9–11]. However, an important aspect of translational medicine is the fusion of therapy and diagnosis to allow the refinement of therapeutic and diagnostic procedures for disease evaluation and treatment. Studies by various research groups including our own demonstrated the emerging potency of NIS as a theranostic gene [13, 28–31]. In a previous study, we showed the general efficacy of MSCs as gene delivery vehicles for NIS under the control of the constitutively active cytomegalovirus (CMV) promoter. Using NIS both as reporter and therapy gene, we demonstrated active MSC recruitment into the tumor stroma of HCC xenografts and a significant therapeutic effect after application of radioiodide [12]. As a refinement of this approach, we then used the RANTES/CCL5 promoter to help limit NIS transgene expression to the tumor environment and thus enhance tumor specificity, based on the fact that the RANTES gene is induced in MSC when they encounter the tumor milieu [32]. Systemic injection of MSCs carrying the RANTES-NIS construct into mice harboring subcutaneous HCC xenografts or colon cancer liver metastases led to a significant tumoral radioiodide accumulation, resulting in a delay in tumor growth and improved animal survival [13, 14].

Hypoxia has been shown to be a common feature of solid tumors and metastases [20, 21, 33]. Clinical studies in patients with various tumor entities clearly demonstrate reduced survival in patients with hypoxic tumors compared to those patients with better oxygenated tumors [34–38]. Since tumor cells in hypoxic regions are often more resistant to chemo- and radiotherapy [24], selective targeting of these cells has become a central focus of research. Several studies reported promising therapeutic effects in various tumor models using hypoxia-responsive promoters combined with gene-directed enzyme prodrug therapies (GDEPT) such as bacterial nitroreductase/CB1954 (enzyme/prodrug), HSV-TK/ganciclovir, cytochrome P450 reductase/RSU1069 and bacterial cytosine deaminase/5-fluorocytosine either by applying viral vectors or after transplanting cancer cells expressing a prodrug-activating enzyme (reviewed by Harada [39]). Similarly, approaches using hypoxia-responsive promoters combined with the cytotoxic protein BAX after viral delivery or hypoxia-dependent E1A expression for hypoxia-targeted virus replication are promising candidates for cancer therapy [39].

In the current study, we evaluated the use of MSCs as delivery vehicles for NIS driven by a synthetic hypoxia-responsive promoter. Following *in vitro* characterization, HIF-NIS-MSCs were systemically injected into mice bearing subcutaneous or orthotopic HuH7 tumors. HIF-NIS-MSCs were shown to specifically home to HCC xenografts and induce HIF-responsive promoter-driven NIS expression, as evidenced by ^123^I-scintigraphy and ^124^I-PET. Tumor-specific radioiodide uptake activity was confirmed by *ex vivo* biodistribution studies. Interestingly, the maximum radioiodide uptake activity of subcutaneous tumors (3.9 ± 0.4% ID/g) was much lower than that observed in orthotopic tumors (6.9 ± 0.9% ID/g). Immunohistochemical analysis of exogenous MSC content in the tumors and NIS expression provided the explanation for this observation: MSC recruitment into subcutaneous xenografts, and, as a direct consequence, NIS expression, was lower than that seen following MSC recruitment and NIS immunoreactivity in orthotopic tumors. Similar observations were made by Garcia *et al.* [40], who described stronger recruitment of MSCs to orthotopic as compared to subcutaneous HuH7 tumors. It has been shown that cancer cells activate the secretion of several cytokines from liver tissue, including TNF-α, interleukin-1 and VEGF [40, 41] – factors that have also been implicated in the government of MSC migration [42]. Similarly, enhanced expression of adhesion molecules such as vascular cell adhesion molecule-1 (VCAM-1) in the liver vasculature has been observed, additionally driving MSC recruitment and integration into intrahepatic tumors [40]. While subcutaneous tumors are generally more hypoxic than orthotopic tumors due to differences in blood supply [43–45] and thus slightly stronger HIF activation was to be expected in the subcutaneous tumors, the number of recruited MSCs seems to be rate-limiting in this setting. Further, exogenous MSC-specific immunostaining was detected in the spleens of mice in both tumor models, while no NIS expression was detected, demonstrating the high tumor-specificity of our hypoxia-targeting strategy.

In line with these pre-therapy *in vivo* and *ex vivo* studies, no significant effects on tumor growth and mouse survival were observed in the subcutaneous xenograft model after application of a therapeutic dose of ^131^I, while intrahepatic tumor-bearing mice showed a strong response to HIF-NIS-MSC/^131^I treatment as evidenced by reduced tumor growth, associated with reduced tumor cell proliferation as well as blood vessel density and tumor perfusion assessed by CEUS. This ultimately resulted in prolonged survival of treated animals compared to control groups. Thus, the tumoral radioiodide uptake in subcutaneous HCCs, which was approx. 50% lower than that observed in the orthotopic model, was not high enough for a therapeutic effect. As MSCs are primarily recruited to perivascular regions before migrating deeper into the tumor tissue and thus into more hypoxic regions, the hypoxia-targeting strategy, although highly tumor-specific, seems to be more dependent on efficient MSC recruitment.

Subcutaneous tumor models are often the first choice for proof of concept studies. However, orthotopic implantation of liver tumors far better reflects the tumor milieu in HCC patients and thus allows a more realistic evaluation of the efficacy of therapeutic approaches. NIS in its function as reporter gene allows repetitive, non-invasive imaging of tumor hypoxia-mediated NIS expression to select patients that benefit most from hypoxia-targeted therapy and also allows the evaluation of therapy response in these patients [46, 47]. In its function as therapy gene, hypoxia-targeted NIS expression facilitates the destruction of tumor cells in hypoxic regions that are generally more resistant to radio- and chemotherapy [24]. Furthermore, due to the crossfire effect of ^131^I, not only hypoxic tumor cells but also normoxic surrounding tumor cells are destroyed by this approach. In addition, our data on tumor perfusion in the orthotopic xenograft model point towards a self-energizing effect of our therapy approach, as NIS-mediated radioiodide therapy results in reduced tumor perfusion and thus in a higher degree of hypoxia, which then induces NIS expression and subsequently increases radioiodide accumulation.

The ^131^I dose used in this study was empirically determined in earlier experiments after consideration of radiation safety, tolerability and efficacy in accordance with the German law for animal protection [48]. For an allometric adaption of the ^131^I dose used in animal studies to humans, the Food and Drug Administration of the United States generated a table with dose conversion factors based on the body surface area of animals and humans [49, 50]. Using these dose conversion factors, the administered ^131^I dose of 1.5 mCi to the mouse translates to 372 mCi for a human being with a body weight of 75 kg, which lies within the dosimetrically determind dose range (300–600 mCi) in patients with advanced metastasized differentiated thyroid cancer [49, 51]. Therefore, the dose administered to mice in this study is within a range that can safely be extrapolated to humans for further clinical trials.

In contrast to the current study, in previous studies using RANTES-NIS-MSCs [13], MSC recruitment and NIS-mediated tumoral radioiodide uptake were sufficient in subcutaneous HCC xenografts to induce a higher tumor-absorbed dose of 44.3 mGy/MBq followed by a significant therapeutic effect of ^131^I. This is due to a stronger activation of the native RANTES promoter in the tumor microenvironment. The RANTES gene is induced by the inflammatory milieu within the tumor stroma, while the HIF-responsive promoter is activated only once the MSCs have migrated into hypoxic regions of the tumor stroma. Importantly, the human RANTES promoter is not activated by hypoxia (unpublished data), thus a hypoxia-associated MSC approach may more efficiently target these important regions of the tumor.

The proof of concept experiments show that tumor hypoxia can be used to trigger the expression of transgenes in engineered MSC. The synthetic hypoxia-inducible promoter used here has the advantage of allowing highly selective monitoring of individual regulatory pathways as evidenced by the selective response to tumor hypoxia. In future studies, we will build on the results presented here to identify a next generation native gene promoter that strongly responds to hypoxia by integrating diverse tissue “stress” signals and may thus show even better efficacy for MSC-directed theranostic NIS expression in diverse tumor settings.

## MATERIALS AND METHODS

### Cell culture

The human HCC cell line HuH7 was authenticated and purchased from JCRB Cell Bank (JCRB 0403, Osaka, Japan) and grown in Dulbecco's Modified Eagle's medium (DMEM; Sigma-Aldrich, St. Louis, Missouri, USA) supplemented with 10% FBS (FBS Superior, Biochrom/Merck Millipore, Berlin, Germany) and 100 U/ml penicillin/100 μg/ml streptomycin (Sigma-Aldrich). Simian virus 40 (SV40) large T antigen immortalized human bone marrow-derived MSCs were established and characterized as described previously [12]. These MSCs show the same differentiation capacity as primary MSCs without the disadvantages of aging and senescence due to the finite ability of self-renewal and allow for the generation of higher cell numbers [52, 53]. MSCs were cultured in RPMI (Sigma-Aldrich) containing 10% FBS and 100 U/ml penicillin/100 μg/ml streptomycin. Both cell lines were maintained at 37°C in 5% CO_2_.

### Plasmid constructs

To establish the expression vector pGL3-HIF-NIS, full length NIS cDNA was removed from the pcDNA3 expression vector (kindly provided by SM Jhiang, Ohio State University, Columbus, OH, USA). The expression vector pGL3-HIF-LUC (a gift from F Grässer, Universitätsklinikum des Saarlandes, Homburg, Germany) was modified to contain a synthetic promoter composed of a minimal thymidine kinase promoter with six HIF-responsive elements driving transgene expression and a CMV controlled Bsr2 blasticidin resistance gene for selection of transfected cells. NIS cDNA was ligated into the pGL3 expression vector using the T4 DNA Ligase (Roche, Basel, Switzerland). For cloning of the pcDNA-ITR-HIF-Cherry plasmid, the HIF-responsive promoter was amplified from the pGL3-HIF-LUC vector and the fluorescent protein mCherry was amplified from the pCAG-Kosak-Cherry vector (a gift from M Rosemann, Helmholtz Center Munich, German Research Center for Environmental Health, Munich, Germany). Using the MultiSite Gateway Pro Plus Kit (Invitrogen Thermo Fisher Scientific, Waltham, Massachusetts, USA), the HIF-responsive promoter was cloned into the pDONR221-P_2_P_5r_ plasmid to obtain the entry vector pENTR221-6xHRE-tk, whereas mCherry was cloned into the pDONR221-P_5_P_2_ plasmid to obtain the entry vector pENTR221-Cherry according to the manufacturer's instructions. To obtain the pcDNA-ITR-HIF-Cherry expression vector, both entry vectors were recombinated with a modified destination vector pcDNA6.2PLITRBlasti-Dest. The resulting pcDNA-ITR-HIF-Cherry plasmid contains the red fluorescent protein mCherry driven by the HIF-responsive promoter, two sleeping beauty transposition sites and a blasticidin resistance gene.

### Stable transfection of MSCs

HIF-NIS-MSCs were generated by stable transfection of WT-MSCs with the pGL3-HIF-NIS expression vector using Lipofectamine with Plus reagent (Invitrogen) according to the manufacturer's recommendations. To obtain HIF-Cherry-MSCs, WT-MSCs were transfected with pcDNA-ITR-HIF-Cherry and the pCMV(CAT)T7-SB100X plasmid (provided from Z Ivics, Max Delbrück Center for Molecular Medicine, Berlin, Germany) which contains the Sleeping Beauty transposase system for transgene insertion into the host cell genome. The MSCs were electroporated at 960 μF and 230 V using a Bio-Rad Gene Pulser (Bio-Rad, Hercules, California, USA). For both plasmids, blasticidin (Invitrogen) was used to differentiate between transfected and untransfected MSCs. NIS-transfected MSCs were analyzed for their NIS-mediated radioiodide uptake activity and mCherry-transfected MSCs were analyzed for mCherry expression using flow cytometry. The stably transfected cell clone with the highest level of radioiodide accumulation or the highest level of red fluorescence, respectively, was used for further experiments.

### Radioiodide uptake assay

To test inducibility of the HIF-responsive promoter, HIF-NIS-MSCs were stimulated with 300 μM of the hypoxia-simulating agent cobalt chloride (Sigma-Aldrich) for 24 h. NIS-mediated ^125^I (PerkinElmer, Waltham, Massachusetts, USA) uptake of WT-MSCs or HIF-NIS-MSCs was measured as described by Spitzweg *et al.* [54]. Radioiodide uptake was normalized to cell viability.

### Cell viability assay

Cell viability was measured by MTT (Sigma-Aldrich, ratio: 1:100) assay. The absorbance of the formazan product was measured at 620 nm using a Sunrise Microplate Absorbance Reader (Tecan, Männedorf, Switzerland).

### Membrane preparation and Western blot analysis

Whole cell lysates from HIF-NIS-MSCs were extracted with M-PER Mammalian Protein Extraction Reagent (Thermo Fisher Scientific). Western blot analysis was performed as described previously [54]. The mouse monoclonal NIS-specific antibody (abcam, Cambridge, UK) was applied at a dilution of 1:133. Protein loading was controlled by reprobing blots with a monoclonal antibody directed against β-actin (Sigma-Aldrich).

### Spheroid invasion assay

For spheroid formation, HuH7 cells were grown on hydrogel poly(2-hydroxyethyl methacrylate) (polyHEMA, Sigma-Aldrich) -coated culture dishes. When spheroids reached a diameter of 400–600 μm, 2.5 × 10^4^ HIF-Cherry-MSCs labeled with 10 μM CellTracker Green CMFDA (5-chloromethylfluorescein diacetate, Life Technologies, Carlsbad, California, USA) were added for 2 h at room temperature (RT). After MSC attachment/invasion, HuH7 spheroids were washed and incubated for 48 h at 37°C. Frozen spheroids were sectioned and fixed with 4% formalin. For fluorescent microscopy, nuclei were counterstained with DAPI (4′,6-diamidino-2-phenylindole) and embedded in 50% glycerol and 0.2% propyl gallate (Sigma-Aldrich) in PBS. Sections were imaged at 20× magnification on a Leica DM IL microscope (Leica Microsystems, Wetzlar, Germany) equipped with a Jenoptik ProgRes CCD camera (Jenoptik, Jena, Germany) and ProgRes CapturePro 2.6 software (Jenoptik). Analysis was performed using ImageJ software (NIH, Bethesda, Maryland, USA).

### Animals

Female CD1 nu/nu mice were purchased from Charles River (Sulzfeld, Germany) and maintained under specific pathogen-free conditions with access to standard nude mouse chow (ssniff, Soest, Germany) and water *ad libitum*. Animals were allowed to acclimatize for one week prior to subcutaneous or intrahepatic tumor cell injections. To reduce thyroidal iodide uptake and consequently maximize tumoral radioiodide uptake, animals were pre-treated with 5 mg/l L-thyroxine (Sigma-Aldrich) in their drinking water for 10 days prior to radioiodide (^123^I, ^124^I, ^131^I) application as described previously [29, 55, 56]. The experimental protocol was approved by the regional governmental commission for animals (Regierung von Oberbayern, Munich, Germany).

### Establishment of subcutaneous and orthotopic HCC xenografts

Subcutaneous HCC xenografts were established in 6-week old mice by injecting 5×10^6^ HuH7 cells resuspended in 100 μl PBS into the flank region. Tumor volumes were measured regularly and estimated using the equation: tumor volume = length × width × height × 0.52. Mice were sacrificed when tumors started to necrotize or exceeded a size of 1500 mm^3^.

To establish orthotopic HCC xenografts, 1×10^6^ HuH7 cells resuspended in 25 μl PBS and 25 μl Matrigel (Corning, Corning, New York, USA) were injected into the liver after laparotomy (adapted from Niess *et al.* [10]) of 7-week old mice. Mice were sacrificed when healthy liver tissue amounted to less than 30% or when mice showed other signs of illness.

### Radioiodide biodistribution studies *in vivo*

As soon as subcutaneous HCC xenografts reached a diameter of 3–5 mm or when intrahepatic HCC xenografts reached a diameter of approx. 3 mm, animals received 5 × 10^5^ HIF-NIS-MSCs (subcutaneous model: *n* = 12; orthotopic model: *n* = 6) or WT-MSCs (subcutaneous model: *n* = 9; orthotopic model: *n* = 2) in 500 μl PBS three times in 5-day-intervals via the tail vein. 72 hours after the last MSC application, 18.5 MBq (0.5 mCi) ^123^I (GE Healthcare, Little Chalfont, UK) or 13 MBq (0.35 mCi) ^124^I (PerkinElmer) were injected intraperitoneally and radioiodide biodistribution was assessed using a gamma camera equipped with a low-energy high resolution (LEHR) collimator (e.cam, Siemens, Munich, Germany) or a Siemens Inveon P120 microPET (Siemens), respectively. In a subset of mice, the competitive NIS inhibitor sodium perchlorate (2 mg/mouse; Sigma-Aldrich; subcutaneous model: *n* = 4; orthotopic model: *n* = 3) was injected intraperitoneally 30 min prior to radioiodide administration to verify that tumoral radioiodide accumulation was NIS-mediated. Gamma camera images were analyzed using HERMES GOLD (Hermes Medical Solutions, Stockholm, Sweden), whereas PET images were reconstructed with the software Inveon Acquisition Workplace (Siemens) and analyzed using Inveon Research Workplace (Siemens). Regions of interest were defined and quantified as % ID/g tumor. The radioiodide retention time within the tumor was determined by serial scanning after radioiodide injection. Dosimetric calculations were done according to the concept of medical internal radiation dose with a RADAR dose factor (http://www.doseinfo-radar.com/).

### Radioiodide biodistribution studies *ex vivo*

For *ex vivo* radioiodide biodistribution analyses, animals were injected with HIF-NIS-MSCs (subcutaneous model: *n* = 12; orthotopic model: *n* = 6) as described above followed by an intraperitoneal injection of 18.5 MBq ^123^I 72 h later. A subset of HIF-NIS-MSC-treated mice was additionally treated with sodium perchlorate (subcutaneous model: *n* = 5; orthotopic model: *n* = 3). 4 h after ^123^I injection, mice were sacrificed and organs of interest were dissected, weighed and ^123^I accumulation was measured in a Packard Cobra Quantum Gamma Counter (GMI, Ramsey, Minnesota, USA). Results are expressed as % ID/g.

### Immunohistochemical analysis of NIS and SV40 large T antigen expression

Immunohistochemical staining of paraffin-embedded or frozen tissue sections derived from HuH7 tumors or other organs of interest was performed as described by Spitzweg *et al.* [57] with a mouse monoclonal NIS-specific antibody (Merck Millipore; 1:1000) for NIS detection or using a M.O.M. Immunodetection Kit (Vector Laboratories, Burlingame, California, USA) with a mouse monoclonal anti-SV40 large T antigen antibody (Calbiochem/Merck, Darmstadt, Germany; 1:2000) for MSC detection. SV40 large T antigen was used for MSC immortalization and could therefore be used to specifically detect exogenously applied engineered MSCs. Sections were imaged at 10× magnification on an Olympus BX41 microscope (Olympus, Shinjuku, Tokio, Japan) equipped with an Olympus XC30 CCD camera (Olympus) and Olympus Cell^A^ software (Olmypus).

### Radioiodide therapy studies *in vivo*

Once subcutaneous HCC xenografts had reached a diameter of 3–5 mm or two weeks after intrahepatic tumor cell injection, mice were injected following the same pattern as described previously [12, 13]. Two groups received 55.5 MBq (1.5 mCi) ^131^I (GE Healthcare) 48 h after the last of three HIF-NIS-MSC (5×10^5^ cells/500 μL PBS; HIF-NIS-MSC + ^131^I; subcutaneous model: *n* = 11; orthotopic model: *n* = 7) or WT-MSC (5×10^5^ cells/500 μL PBS; WT-MSC + ^131^I; subcutaneous model: *n* = 9; orthotopic model: *n* = 5) applications in 2-day-intervals. This treatment cycle was repeated once 24 h after the last ^131^I injection. 24 h after these two treatment cycles one additional MSC application was administered followed by a third ^131^I injection 48 h later. The third group was treated with saline instead of ^131^I after injection of HIF-NIS-MSCs (HIF-NIS-MSC + NaCl; subcutaneous model: *n* = 13; orthotopic model: *n* = 5).

### CEUS

Tumor growth and tumor blood flow of orthotopic HCC xenografts were monitored by CEUS as described by Eichhorn *et al.* [58]. CEUS was performed on an Acuson Sequoia 512 (Siemens) combined with a 15L8W ultrasound probe using the Cadence contrast pulse sequencing technology. To assess tumor volume, tumors were imaged in longitudinal and transverse direction in brightness-mode with a frequency of 14 MHz and a mechanical index of 0.2 or less. After scanning through the tumor, electronic calipers were used to measure the tumor's maximum cross-sectional dimensions. Tumor volumes were estimated using the equation: tumor volume = length × width × height × 0.52. For the evaluation of tumor blood flow, the contrast agent SonoVue (Bracco, Milano, Italy) was applied via a tail vein catheter and perfusion was recorded on digital cine clips before and up to 1 min after the application of the contrast agent at a frame rate of 8–10 Hz. Digital cine clips were exported in a Digital Imaging and Communications in Medicine (DICOM) format for off-line analysis with VueBox (Bracco Suisse, Geneve, Switzerland) using a bolus kinetic model [59]. To this end, a region of interest was drawn around the entire tumor of each animal. The contrast agent concentration was estimated using pre-defined calibration curves [59] and the perfusion-related parameters PE, WiAUC, WiR and WiPI were estimated.

### Immunofluorescence analysis of CD31 and Ki67

Frozen tissue sections were fixed in methanol/acetone and blocked in 12% bovine serum albumin (BSA)/PBS at RT for 30 min prior to the application of the primary rabbit polyclonal antibody against Ki67 (abcam; 1:200) and the rat monoclonal antibody against CD31 (BD Pharmingen, Heidelberg, Germany; 1:200). Sections were then incubated with a secondary anti-rabbit Alexa488-conjugated antibody (Jackson ImmunoResearch, West Grove, Pennsylvania, USA) for Ki67 staining and secondary anti-rat Cy-3-conjugated antibody (Jackson ImmunoResearch) for CD31 staining along with Hoechst bisbenzimide (5 μg/ml) to counterstain nuclei. Sections were embedded using Fluorescent Mounting Medium (Dako, Hamburg, Germany) and imaged at 10× magnification on an Axiovert 135 TV fluorescence microscope (Carl Zeiss, Munich, Germany) equipped with an AxioCam MRm CCD camera (Carl Zeiss) and AxioVision Rel. 4.8 software (Carl Zeiss). Immunostainings were captured at identical illumination conditions, exposure time and system settings for digital image processing. Quantification of cellular proliferation (percentage of Ki67 positive cells in the tumor) and blood vessel density (percentage of CD31 positive area in the tumor) was performed by evaluation of 4–5 visual fields per tumor using ImageJ software (NIH).

### Statistical methods

Values are reported as mean ± SEM or, for survival plots, percent. Statistical significance was tested by two-tailed Student's *t*-test or by Mann-Whitney *U* test for survival curves. *p* values < 0.05 were considered significant (*p < 0.05; **p < 0.01; ***p < 0.001).
